# Does trunk muscle strength affect spinal deformity in adult female patients?: Evaluation of cross sectional area of psoas major and lumbar extensor muscles

**DOI:** 10.1186/1748-7161-10-S1-P13

**Published:** 2015-01-19

**Authors:** Kei Watanabe, Masayuki Ohashi, Toru Hirano, Keiichi Katsumi, Noriaki Yamamoto, Naritoshi Sato, Naoto Endo

**Affiliations:** 1 Department of Orthopaedic Surgery, Niigata University Medical and Dental General Hospital, Japan; 2 Department of Orthopaedic Surgery, Niigata Rehabilitation Hospital, Japan; 3 Department of Physical Therapy, Niigata University of Health and Welfare, Japan

## Objective

The purpose of this study was to evaluate cross sectional area (CSA) of psoas major and lumbar extensor muscles, and investigate the relation between truck muscles and spinal deformity in female patients with adult spinal deformity (ASD).

## Material and methods

Twenty-five female patients with ASD (mean age 64.3 years) who underwent fusion surgery were included for analysis. Diagnosis was lumbar scoliosis in 10 patients, kyphoscoliosis in 11, and kyphosis in 4. Radiographic measurement of spinal alignment and CSA of lumbar trunk muscles including psoas major and lumbar extensor muscles measured by Magnetic Resonance Imaging (MRI) were evaluated. The CSAs of trunk muscles were measured at the cranial endplate of L4 vertebra for a comparative estimation of muscle size. A negative value indicated lordosis in evaluation of spinal alignment.

## Results

CSA of the psoas major adjusted by mean height was 546.2mm^2^ (296~769.3), and that of the lumbar extensor was 1389.9mm^2^ (913.6~2518.4). The mean radiographic parameters showed as follows; C7 plum line: 5.47mm, major Cobb angle: 40.0º, SVA: 93.0mm, upper thoracic kyphosis (T2-5): 8.1º, thoracic kyphosis (T5-12): 13.9º, thoracolumbar kyphosis (T10-L2): 10.6º, lumbar lordosis (L1-S1): -8.5º, pelvic incidence: 53.2º, pelvic tilt: 36.6º, and sacral slope: 16.6º. The CSA of psoas major was correlated with thoracolumbar kyohosis (rs=0.562, p<0.01), and pelvic tilt (rs=-0.481, p<0.05). The CSA of lumbar extensor was correlated with lumbar lordosis (rs=-0.533, p<0.05), and pelvic tilt (rs=-0.433, p<0.05). The CSAs of trunk muscles demonstrated no correlations with age, and body mass index.

## Conclusions

There are significant correlation between lumbo-pelvic alignment and CSAs of trunk muscles including psoas major and lumbar extensor muscles. The trunk muscles may play an important role in supportive tissue of lumbar spine and pelvis, and affect progression of adult spinal deformity.

